# Development of a Liquid Chromatography/Mass Spectrometry-Based Inhibition Assay for the Screening of Steroid 5-α Reductase in Human and Fish Cell Lines

**DOI:** 10.3390/molecules26040893

**Published:** 2021-02-08

**Authors:** Dahye Kim, Hyunki Cho, Ruth Eggers, Sang Kyum Kim, Chang Seon Ryu, Young Jun Kim

**Affiliations:** 1 Environmental Safety Group, Korea Institute of Science and Technology (KIST) Europe Forschungsgesellschaft mbH, 66123 Saarbruecken, Germany; da-hye.kim@daum.net (D.K.); hyunki.cho@kist-europe.de (H.C.); rutheggers@gmx.de (R.E.); youngjunkim@kist-europe.de (Y.J.K.); 2 College of Pharmacy, Chungnam National University, Daejeon 34134, Korea; sangkim@cnu.ac.kr; 3 Division of Energy & Environment Technology, University of Science and Technology, Daejeon 34113, Korea

**Keywords:** 5α-reductase inhibitors, dihydrotestosterone, in vitro, dutasteride, finasteride, adverse outcome pathway

## Abstract

Steroid 5-α reductase (5AR) is responsible for the reduction of steroids to 5-α reduced metabolites, such as the reduction of testosterone to 5-α dihydrotestosterone (DHT). A new adverse outcome pathway (AOP) for 5AR inhibition to reduce female reproduction in fish (AOP 289) is under development to clarify the antiestrogenic effects of 5AR inhibitors in female fish. A sensitive method for the DHT analysis using chemical derivatization and liquid chromatography–tandem mass spectrometry was developed. A cell-based 5AR inhibition assay that utilizes human cell lines, a transient overexpression system, and fish cell lines was developed. The measured IC_50_ values of two well-known 5AR inhibitors, finasteride and dutasteride, were comparable in the different systems. However, the IC_50_ of dutasteride in the fish cell lines was lower than that in the human cell lines. Finasteride showed a higher IC_50_ against the RTG-2 cell line. These results demonstrated that 5ARs inhibition could differ in terms of structural characteristics among species. The assay has high sensitivity and reproducibility and is suitable for the application in 5AR inhibition screening for various endocrine disruption chemicals (EDCs). Future studies will continue to evaluate the quantitative inhibition of 5AR by EDCs to compare the endocrine-disrupting pathway in different species.

## 1. Introduction

Steroid 5-α reductase (5AR, EC. 1.3.99.5) is a membrane-bound protein that is responsible for reducing steroids such as testosterone, progesterone, and androstenedione to 5-α reduced metabolites such as 5-α dihydrotestosterone (DHT), 5-α dihydroprogesterone and androstanedione, respectively. There are three isoforms of 5AR in humans: SRD5A1, SRD5A2, and SRD5A3. SRD5A1 and SRD5A2 have functionality for 5-α reduction of steroids in humans. DHT is a more potent androgen than testosterone and has a function in androgen receptor activation [1,2,3]. The regulation of 5AR is important for the treatment of benign prostate hyperplasia (BPH) and prostate cancer (PC), and 5AR inhibitors have also been used for the treatment of baldness [4,5,6].

5AR inhibition was suggested as a new molecular initiating event (MIE) in the adverse outcome pathway (AOP) 289 [7]. AOP 289, which is entitled ‘Inhibition of 5α-reductase leading to impaired fecundity in female fish’, describes the effects of 5AR on reducing estradiol and further decreasing egg production via vitellogenin reduction. 5AR is expressed in both sexes, and DHT is involved in estradiol (E2) level regulation [8]. Even though a lower expression of 5AR was detected in females, its inhibition reduced the fecundity of fish and affected several aspects of reproductive endocrine functions in both sexes of fathead minnows [9]. For the development of a quantitative AOP for 5AR inhibition, a quantitative structure–activity relationship is required for endocrine disruption chemical (EDC) evaluation. Several methods have been described for screening the pharmacological aspects of 5AR inhibitors, but experimental data are limited in fishes for screening for endocrine disruption.

In practice, 5AR inhibition studies are traditionally conducted using radioactive substrates with thin layer chromatography or high-performance liquid chromatography (HPLC) detection [10,11]. A native substrate method without radiolabeled isotopes that utilizes a spectrophotometric method [12] and a HPLC-UV detection method was also developed [13]. However, these methods have not been extensively applied due to their limitations, which include safety issues with radiometric assays and low sensitivity. The liquid chromatography-tandem mass spectrometry (LC-MS/MS) method can be used for high-throughput screening (HTS) techniques, and combinational chemistry during drug discovery and development has led to a tremendous increase in the number of compounds to be evaluated for potential 5AR inhibition [14,15]. Recently, sensitive chemical derivatization methods for DHT detection in LC-MS/MS were developed [16].

In the present study, using this chemical derivatization technique, a cell incubation method was developed, and the metabolites of the substrates were determined in a single assay using LC-MS/MS for HTS of 5AR inhibition. LNCaP clone FGC (LNCaP) and DU-145 cells that express the SRD5A1 gene, SW-13 cells that express the SRD5A1 and SRD5A2 genes, and HEK-293 cells with transient overexpression of the SRD5A1 and SRD5A2 genes were compared to establish the enzyme inhibition method. In addition, to understand species differences in 5AR between fish and humans, the inhibition of 5AR was compared in the 5AR-expressing zebrafish liver cells (ZFL) and rainbow trout gonad cell lines (RTG-2).

## 2. Results

### 2.1. Method Validation

#### 2.1.1. Linearity of the Calibration Curve and LLOQ

**Figure 1:**
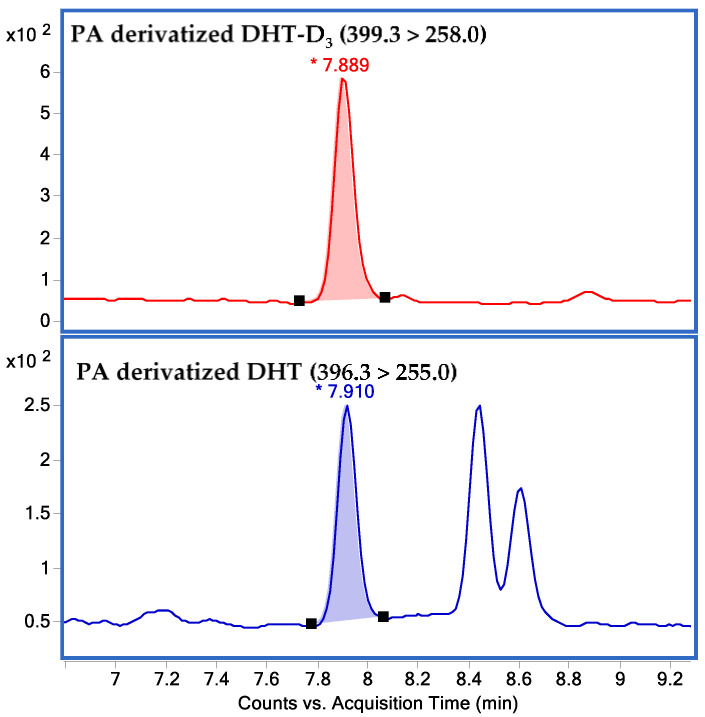
Chromatograms of 2-picolinic acid (PA)-derivatized 5-α dihydrotestosterone (DHT) and DHT-D3.

The 1/x weighted linear regression calibration curve for DHT was obtained by plotting the MRM peak area ratio (analyte/IS) versus the concentration over the working range 0.01–1000 nM for the assay media. The 1/x weighted linear correlation coefficient (R2) for DHT exceeded 0.995. The LLOQ of this method for DHT was 0.05 nM. Chromatograms of 2-picolinic acid (PA)-derivatized DHT and DHT-d3 are presented in Figure 1.

#### 2.1.2. Accuracy and Precision

The method accuracy and precision that were determined using the low QC, medium QC and high QC samples are presented in Table 1. The inter day accuracies for the low, medium, and high QC samples were 102.3, 104.0, and 95.0%, respectively, and the intraday accuracies for the low, medium, and high QC samples were 101, 98.9, and 95.5%, respectively. The interday precisions were 1.3% for low QC, 0.7% for medium QC, and 1.6% for high QC, and the intraday precisions were 0.9% for low QC, 2.5% for medium QC, and 1.3% for high QC. Acceptable method accuracies and precisions on the QC samples were obtained.

### 2.2. Assay Application in Human Cell Lines

**Figure 2:**
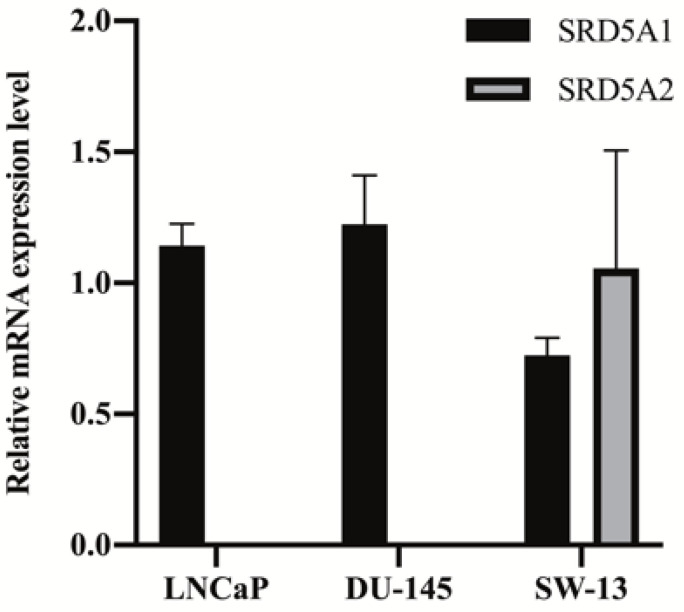
Quantitative PCR analysis for measuring the mRNA expression levels of SRD5A1 and SRD5A2 in the LNCaP, DU-145, and SW-13 cell lines. The data are expressed as the mean ± standard deviation (SD) of three repeated experiments.

**Figure 3:**
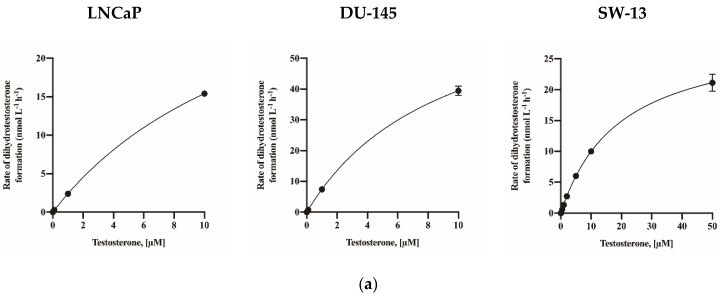

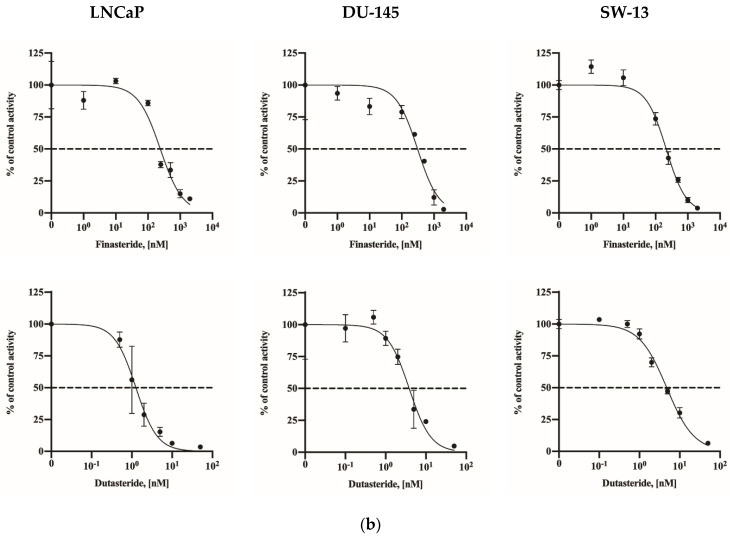
Activity of 5α-reductase (**a**) and inhibitory effects of finasteride and dutasteride (**b**) on LNCaP, DU-145, and SW-13 cells. The data are expressed as the mean ± standard deviation (SD) of three repeated experiments.

The gene expression levels of SRD5A1 and SRD5A2 in LNCaP, DU-145, and SW-13 cells are presented in Figure 2. All cell lines showed SRD5A1 expression, but SRD5A2 expression was identified only for SW-13 cells. For the calculation of K_M_, testosterone treatment was applied in increments of 0 to 10 µM in the LNCaP and DU-145 cells and in increments of 0 to 50 µM in the SW-13 cells for 3 h. The de novo synthesized DHT levels were measured (Figure 3a). The calculated values of K_M_ and V_max_ are presented in Table 2. The V_max_ value of the DU-145 cells was 75.55 nmol/L/h, which exceeded those of the other two cell lines. Based on the calculated K_M_ value as the substrate concentration, inhibition assays were conducted by treating the cells with a selective SRD5A2 inhibitor, namely, finasteride, and a dual SRD5A1 and SRD5A2 inhibitor, namely, dutasteride (Figure 3b). The IC_50_ value of each inhibitor was calculated and is presented in Table 3.

### 2.3. Assay Application in SRD5A2-Overexpressing HEK-293 Cells

**Figure 4:**
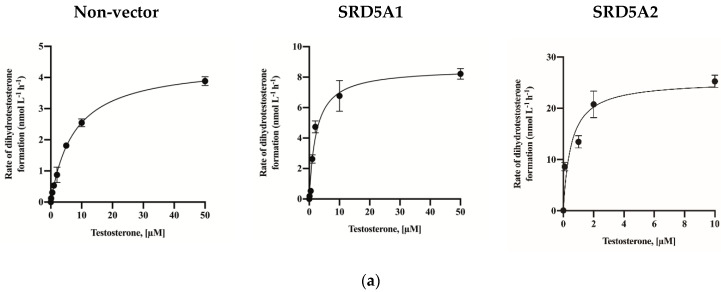

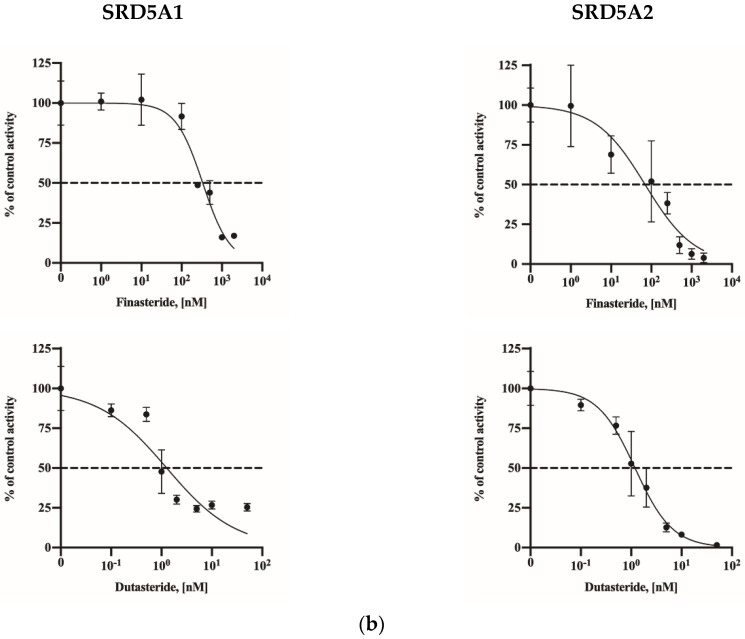
Activity of 5α-reductase (**a**) and inhibitory effects of finasteride and dutasteride (**b**) on transfected HEK-293 cells. The data are expressed as the mean ± standard deviation (SD) of three repeated experiments.

For the calculation of K_M_, testosterone was added in increments of 0 to 50 µM to non-vector- and SRD5A1-HEK293 cells and in increments of 0 to 10 µM to SRD5A2-HEK293 cells 24 h after transfection. The DHT levels were measured (Figure 4a). The calculated K_M_ and V_max_ values are presented in Table 2. SRD5A2 showed a higher production rate (V_max_ and K_M_ were 22.52 and 0.36 nM, respectively) due to the higher affinity of the enzyme for testosterone. Based on the calculated K_M_ values, inhibition assays were conducted by treating the cells with selective SRD5A2 inhibitor finasteride and the dual SRD5A1 and SRD5A2 inhibitor dutasteride (Figure 4b). The IC_50_ values were calculated (Table 3).

### 2.4. Assay Application in a Fish Cell Line

**Figure 5:**
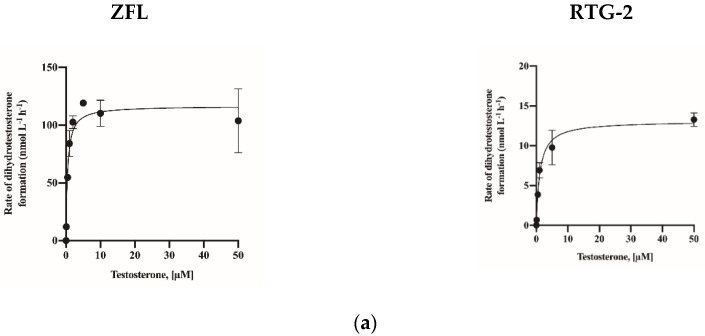

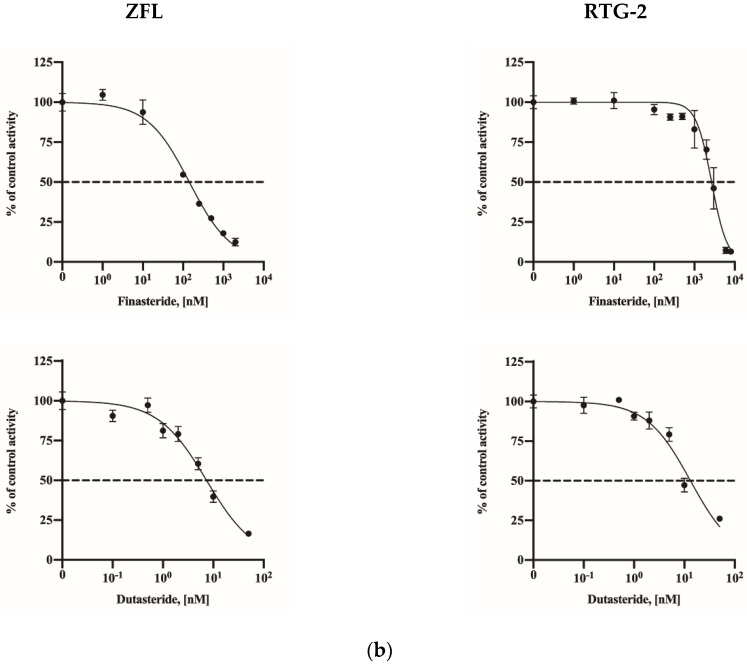
Activity of 5α-reductase (**a**) and inhibitory effects of finasteride and dutasteride (**b**) on and zebrafish liver cells (ZFL) and rainbow trout gonad (RTG-2) cells. The data are expressed as the mean ± standard deviation (SD) of three repeated experiments.

For the calculation of K_M_ for optimized assay conditions, ZFL and RTG-2 cells were treated with testosterone in increments of 0 to 50 µM. The DHT levels were measured (Figure 5a). The calculated K_M_ and V_max_ values are presented in Table 2. Based on the calculated K_M_ values, inhibition assays were conducted (Figure 5b). Both the RTG-2 and ZFL cells showed lower K_M_ values than human cell lines. The V_max_ value of the ZFL cells (116.6 nmol/L/h) substantially exceeded those of the 5AR-overexpressing cell line and the human cell lines (29.35 in LNCaP, 75.55 in DU-145, and 29.35 in SW-13). The IC_50_ value of finasteride in the RTG-2 cells was 2459 nM, which exceeded those of the 5AR-overexpressing cell line and the human cell lines (1.27 in SRD5A1 and 1.16 in SRDA2-overexpressing HEK cells). Furthermore, the IC_50_ values of dutasteride in both fish cell lines exceeded those of the 5AR-overexpressing cell line and the human cell lines (Table 3).

## 3. Discussion

Fluorinated anhydride acylation methods are widely used for gas chromatography mass spectrometry (GC-MS) for steroid quantification. Similar to the acylation reaction of fluorinated anhydrides and the hydroxyl group of the seventeenth carbon position in the steroid reaction, derivatization using PA showed a higher sensitivity in the detection of 17-OH steroids, such as corticosteroids, in ESI-LC/MS [17]. Recently, LC-MS based quantification methods for androgens such as DHT that utilize various sample sources were developed, and 5AR inhibition studies were conducted [16,18,19,20,21,22]. The method in the present study requires an additional derivatization step compared to the direct measurement. However, compared to these reports, the LLOQ of DHT (14.5 pg/mL) in the present study showed higher sensitivity than hydroxylamine hydrochloride derivatization [18] or direct measurement [19,23] by using LLE after PA derivatization. Also, methods using solid-phase extraction have been developed for the detection of steroids, but these methods are not efficient in time and cost -effective compared with liquid-liquid extraction [16,24,25]. The present study also used 2 times the liquid-liquid extraction step using MTBE after and before derivatization, this process increased the recovery of target compounds from 69 to 74% to 89–108% [16]. The lower limit of quantification of other studies using spectrophotometric method for DHT were from 0.2–10 nM [12,22,26], and other studies using radioactive substrates were range of 25 to 250 ng. The comparison study between immunoassay and LC/MS detection of DHT showed that the variation of detection was relatively more significant in immunoassay than in MS systems [27]. Thus, the method in the present study has an advantage for the detection of DHT than other methods.

A cell-based assay has additional factors that need to optimizing assay condition, but it has more reliability to in vivo system than purified enzyme or centrifuged fraction. Inhibition of 5AR reduced the DHT levels in tissues and can affect the androgen receptor (AR) expression [28,29,30]. Steroids such as androgens, estrogens and corticosteroids and inhibitors of 5AR are widely utilized in pharmacological applications, and these chemicals may act as EDCs and substantially impact fish and other species that are exposed to the environment [31,32]. We compared the 5AR activities and inhibition rates of 5AR by finasteride and dutasteride between human cell lines and fish cell lines. The V_max_ and K_M_ values in human cell lines were the largest in the DU-145 cells (Table 2). This result may be related to AR signaling. The LNCaP cell line was AR-positive, whereas the DU-145 cell line was AR-negative. DHT can be metabolized to DHT-glucuronide by the uridine diphosphate-glucuronosyltransferase (UGT) 2B15 and 2B17 enzymes in prostate cells, and these enzymes are modulated by AR [33]. It is possible that the rate of DHT production in AR-negative DU-145 cells exceeds those in other cell lines. The optimal pH of SRD5A1 activity is a broad range from 6.0 to 8.5, and the range for SRD5A2 is from 5.0 to 5.5 [12,33,34]. The steroid affinity of SRD5A2 is 10–20 times higher than that of SRD5A1 under optimal conditions [35].

Under transient transfection conditions, the V_max_ values in HEK-293 cells that were transfected with SRD5A1 and SRD5A2 were approximately 2 times and 5 times larger, respectively, than those of the nontransfected HEK-293 cells. The K_M_ values in HEK-293 cells that were transfected with SRD5A1 and SRD5A2 were approximately 3.3 times and 21 times smaller, respectively. The transfected cell lines did not show a higher V_max_ compared to human cell lines, but the K_M_ values decreased; hence, we assume that transient conditions can be used for the comparison of specific enzyme inhibition.

Both fish cell lines were more sensitive to testosterone treatment than human cell lines, and the ZFL cells were more sensitive than the RTG-2 cells. Other studies showed that the activity of 5AR in goldfish (*Carassius auratus*) was high in nonreproductive tissues such as the liver, brain and pituitary tissues, and it was reported that the expression pattern of SRD5A2 in toadfish (*Opsanus tau*) was significantly higher in the liver than in the gonad, in contrast to that in humans [36,37]. In the case of rainbow trout (*Oncorhynchus mykiss*), SRD5A activity was confirmed in the skin of males and females [38,39]. Although we did not measure the 5AR activity in whole tissue cells, our results demonstrated that fish cell lines are more sensitive to testosterone than human cell lines. The results showed a clear difference in steroid metabolism between the human and fish cell lines. In addition, the activity of 5AR in fish liver cells exceeded that in gonad cells.

The results of the 5AR inhibition assay demonstrated that dutasteride was more potent than finasteride in all cell lines. This is because dutasteride, which is a 5AR dual inhibitor, had a higher 5AR inhibition efficiency, and this tendency was similar to that observed in previous studies [39,40]. However, all the fish cell lines except ZFL on finasteride showed relatively lower sensitivity than human cell lines, and the IC_50_ value of RTG-2 on finasteride was 14 times larger than those on other cell lines. The IC_50_ values of dutasteride in fish cell lines exceeded those in human cell lines.

Similar to our results, other studies also reported that the activity of inhibitors differs among species. The inhibitory effects of finasteride, which mainly inhibits SRD5A2, were similar among dogs, monkeys, and humans, whereas finasteride inhibited both SRD5A1 and SRD5A2 in rats [41,42]. In addition, in a comparison of rat and human IC_50_ values comparisons of finasteride using rat 5α-reductase in prostate microsome were 11 nM, 13 nM, and 237 nM, and IC_50_ values of dutasteride to rat and human 5α-reductase were in the range of 0.2–7 nM [14,43,44,45,46,47]. It was suggested that the difference in amino acid sequences may present a differential response to inhibitors [42]. The amino acid sequence identity of SRD5A1 in humans and fish was approximately 50.2–51.7%, and for SRD5A2 the amino acid identity was detected as 42.4–52.3% (Table 4). Due to the difference in amino acid sequences, the enzymes may differ structurally, and accordingly, the interactions between the substrate or inhibitor and the enzymes can also differ. This suggests that known EDCs may exert various adverse effects on several species through other interactions; thus, future studies are necessary for identifying differences in the impact of EDCs among species.

## 4. Materials and Methods

### 4.1. Chemicals and Reagents

Fetal bovine serum (FBS), Leibowitz’s L-15 medium, the Roswell Park Memorial Institute (RPMI) 1640 medium, Ham’s F12 medium, Eagle’s minimal essential medium (EMEM), Dulbecco’s modified Eagle’s medium (DMEM), the Opti-MEM medium, a penicillin/streptomycin solution and trypsin were obtained from GIBCO (Grand Island, NY, USA). Trout serum was purchased from Caisson Laboratories (Smithfield, VA, USA). Mouse epidermal growth factor (EGF) and HEPES were purchased from Thermo Fisher Scientific (Waltham, MA, USA). Sodium bicarbonate, bovine insulin, DHT, DHT-D3 solution, methyl-tertiary-butyl ether (MTBE), trimethylamine (TEA), tetrahydrofuran (THF), 2- PA, 4-(dimethylamino) pyridine (DMAP), 2-methyl-6-nitrobenzoic anhydride (MNBA), and acetic acid were purchased from Sigma-Aldrich (St. Louis, MO, USA), and HPLC-grade formic acid was purchased from Fisher Scientific (Pittsburgh, PA, USA). MS-grade methanol and water were obtained from VWR (Westchester, NY, USA). The stock solution and internal standard were prepared in methanol. The derivatization reagent was prepared by dissolving 25.0 mg of PA, 10.0 mg of DMAP, and 20.0 mg of MNBA in 1 mL of THF (Yamashita et al., 2009) and vortexing. Then, the mixture was left at room temperature for at least 5 min before the sample pretreatment.

### 4.2. Cell Culture

HEK-293, LNCaP, DU-145, SW-13, and ZFL cell lines were obtained from the American Type Culture Collection (ATCC; Manassas, VA, USA) and cultured according to their instructions. The HEK-293 cells were cultured in a high-glucose DMEM that contained 10% FBS, 100 units/mL penicillin, and 100 µg/mL streptomycin. The DU-145 cells were cultured in EMEM that contained 10% FBS, 100 units/mL penicillin, and 100 µg/mL streptomycin. The LNCaP cells were cultured in RPMI 1640 that contained 10% FBS, 100 units/mL penicillin, and 100 µg/mL streptomycin at 37 °C in 5% CO_2_. SW-13 cells were cultured in Leibovitz’s L-15 medium with 10% FBS at 37 °C without CO_2_. ZFL cells were cultured in a complete medium that was composed of 50% L-15, 35% DMEM medium, and 15% F12 medium that contained 0.15 g/mL sodium bicarbonate, 15 mM HEPES, 0.01 mg/mL bovine insulin, 50 ng/mL mouse EGF, 5% FBS, and 0.5% trout serum at 28 °C without CO_2_. RTG-2 cells were obtained from Prof. Kristin Schirmer (EAWAG, Switzerland) and cultured in the L-15 medium with 5% FBS, 100 units/mL penicillin, and 100 µg/mL streptomycin at 20 °C without CO_2_.

### 4.3. Transient Overexpression

SRD5A1 and SRD5A2 expression vectors were purchased from GenScript (pcDNA3.1+/C-(K)-DYK-SRD5A1, OHu02727D, and pcDNA3.1+/C-(K)-DYK-SRD5A2, OHu18065D, respectively). Transient overexpression was induced using transfection of cDNA with lipofectamine (Thermo Fisher Scientific, Waltham, MA, USA). HEK-293 cells were seeded in 24-well plates at a density of 10^5^ cells per well and incubated at 37 °C in an atmosphere of 5% CO_2_. After overnight culture, 500 ng of cDNA and 0.75 µL of the Lipofectamine 3000 reagent were diluted in the Opti-MEM medium and incubated for 15 min for DNA-lipid complex formation. The DNA-lipid complex was added to the wells and incubated for 6 h. After incubation, the sample-treated medium was changed to the complete culture medium and incubated for 18 h.

### 4.4. Cell Culture Assay Application

All cells were seeded on a 24-well plate. The seeding densities of the DU-145, LNCaP, and SW-13 cells were 0.5 × 10^5^ cells per well. The ZFL and RTG-2 cells were seeded at densities of 1.0 × 10^5^ cells and 2.0 × 10^5^ cells, respectively. After overnight culture, the culture media was aspirated from each well and treated with testosterone that was diluted in the complete medium for 3 h and 6 h. In the case of transiently transfected HEK-293 cells, the testosterone treatment was applied after transient overexpression under the same conditions as other cell lines. The treated media were collected from each well and centrifuged at 3000× *g* for 5 min at 4 °C. The supernatants were stored at −80 °C until needed. A selective SRD5A2 inhibitor, namely, finasteride, and a dual inhibitor of SRD5A1 and SRD5A2, namely, dutasteride, were used as inhibitors of 5-α reductase. The seeding conditions of all cells were the same as those previously described. After overnight culture, the culture medium was aspirated, and the cells were cotreated with a medium that contained testosterone and inhibitors for 3 h. The medium was collected from each well and centrifuged at 3000× *g* for 5 min at 4 °C. The supernatants were stored at −80 °C until analysis.

### 4.5. qRT PCR

The total RNA was isolated using a column-based kit (Qiagen, Valencia, CA, USA). cDNA was synthesized from 500 ng of the total RNA using a high-capacity RNA-to-cDNA kit (Applied Biosystems, Foster City, CA, USA) according to the manufacturer’s instructions. qRT-PCR assays were conducted using a TaqMan gene expression assay on a 7500 FAST real-time PCR system (Applied Biosystems). The TaqMan assay ID is as follows (Gene, assay ID): RPLO0, Hs00420895_gH; SRD5A1, Hs00165843_m1; SRD5A2, Hs00165843_m1.

### 4.6. Sample Preparation

A method that was modified by [15] was used for DHT extraction from the samples. Each sample, which included the calibration, QC, and assay medium, was placed in 1.5 mL PP tubes and spiked with a 0.5 ng/mL DHT-D3 internal standard prior to extraction. All sample tubes were vortexed for 5 s, and the samples were extracted using a liquid-liquid extraction (LLE) method via the addition of 600 µL of MTBE. The samples were vortexed and centrifuged at 4500× *g* rpm for 5 min, and the organic phase was transferred into glass tubes. The extraction step was repeated once, and the organic phase extracts were dried under a stream of nitrogen. After the samples were dried, 100 µL of the derivatization reagent and 100 µL of TEA were added for DHT derivatization. The samples were vortexed and incubated at room temperature, and 1 mL of 10% acetic acid was added to stop the reaction after 30 min of incubation. The LLE step, which was conducted before the derivatization step, was repeated twice. The organic phase extracts were collected, dried under a stream of nitrogen, and reconstituted in 50 µL of 80% methanol that contained 0.1% formic acid for LC-MS/MS analysis.

### 4.7. Instrumental Conditions

The extracts were analyzed for DHT via ultra-performance LC-MS/MS (Agilent 1200/6460C QQQMSD coupled Jet Stream technology electrospray ion (ESI) source; Agilent Technologies, Santa Clara, CA, USA). To separate the analytes, a Kinetex XB-C18 column (2.1 mm × 150 mm, 2.6 μm) that was fitted with a ZORBAX Eclipse Plus C18 guard column (2.1 mm × 5 mm, 1.8 μm) was used. The mobile phase solvents were 0.1% formic acid and methanol, with a flow rate of 300 µL/min for 14 min and a sample injection volume of 10 µL. The gradient started at 5% methanol, was increased to 90% with a 3 min ramp, and was maintained until 5 min. Then, the ramp was increased to 95% methanol until 13 min. At 13.1 min, the ramp was decreased to 5% methanol, which was maintained until 14 min. Mass spectrometry was conducted in the positive ion electrospray mode and multiple reaction mode (MRM) to identify and quantify DHT. The MRM transitions are 396.3 > 255.0 and 273.0 for PA-derivatized DHT and 399.3 > 258.0 and 276.0 for DHT-D3, respectively. The optimized MS conditions are as follows: gas temperature of 350 °C, gas flow of 10 L/min, nebulizer gas pressure of 45 psi, sheath gas temperature of 350 °C, sheath gas flow of 11 L/min, capillary voltage of 3500 V, nozzle voltage of 500 V, and collision energies of 16 V for DHT and 14 V for DHT-D3.

### 4.8. Calibration Curve and LLOQ

A linear calibration curve was established using a standard solution that consisted of a concentration series of 0.1, 0.5, 1, 5, 10, 50, 100, 500, and 1000 nM DHT with 5 ng/mL DHT-D3. The calibrators for DHT were prepared in an assay medium with a blank (which contained only 5 ng/mL DHT-D3). To evaluate the linearity of the calibration curve, a 1/x weighting linear regression was used. The LLOQ was defined as the lowest concentration of the calibrators at which the signal sensitivity was 3-fold higher than those of the corresponding blank samples.

### 4.9. Accuracy and Precision

The accuracy and precision of the method were evaluated using intra- and interday quality control (QC) samples. Five replicates each of low QC, medium QC, and high QC samples were prepared by spiking into standard solutions of DHT and DHT-D3 in an assay medium. Their concentrations are 5, 50, and 500 nM, respectively, which represent 100% DHT accuracy of each QC set. The method accuracy was evaluated based on the recoveries (%) that were calculated for each QC spiking level. The precision of the method was expressed as the coefficient of variation (CV, %). CV was determined by dividing the relative standard deviations of the QC samples by the average DHT concentration of the QC samples. The interday accuracy and precision were determined via three parallel analyses of three sets of QC samples (low, medium, and high). The intraday accuracy and precision were determined via analysis of five replicate samples of each QC set for 3 consecutive days.

### 4.10. Data Analysis

The LC-MS/MS data were analyzed with the MassHunter quantitative analysis software (Agilent). The DHT inhibition in the presence of inhibitors was expressed as a percentage of the corresponding control value. Each point was expressed as the mean ± S.D. A sigmoid-shaped curve was fitted to the data, and the enzyme kinetic module and inhibition parameter IC_50_ were calculated by fitting the Hill equation to the data using nonlinear regression (least-squares best fit modeling) of the plot of the percent control activity vs. concentration of the test inhibitor using GraphPad Prism 8 (GraphPad Software Inc., San Diego, CA, USA). Control samples (without the inhibitor) were assayed in each analytical run. The amount of metabolite in each sample (relative to the control samples) was plotted vs. the inhibitor concentration.

## 5. Conclusions

The present study established cell-based 5AR inhibition assay models using quantitative LC-MS/MS analysis. Using this method, all the fish cell lines except the ZFL cell line for finasteride showed significantly higher IC_50_ values for dutasteride and finasteride. This method can be used as a tool for 5AR inhibitor screening in the early stages of drug discovery. In future studies, the inhibitory potency of chemicals will be evaluated for predicting endocrine disruption via a 5AR inhibition assay to develop quantitative AOPs for 5AR inhibition in fishes.

## Figures and Tables

**Table 1:** Method accuracy and precision (*n* = 5).

	Low QC	Medium QC	High QC
CV%—inter day ^a^	1.3	0.7	1.6
CV%—intra day ^b^	0.9	2.5	1.3
Accuracy%—inter day	102.3	104.0	95.0
Accuracy%—intra day	101.0	98.9	95.5

^a^ Coefficient of variation within days; ^b^ Coefficient of variation between 3 consecutive days.

**Table 2:** V_max_ and K_M_ value for testosterone in each cell line.

		V_max_ (nmol L^−1^ h^−1^)	K_M_ (nM)
Human cell lines	LNCaP	38.67 (34.36–45.16) *	15.10 (12.35–19.25)
	DU-145	75.55 (66.61–90.45)	9.15 (7.01–12.81)
	SW-13	29.35 (27.94–30.90)	19.42 (17.31–21.88)
Overexpression lines	Non vector	4.473 (4.29–4.66)	7.56 (6.74–8.46)
	SRD5A1	8.584 (7.86–9.35)	2.29 (1.70–3.09)
	SRD5A2	22.52 (20.01–25.42)	0.36 (0.18–0.65)
Fish cell lines	ZFL	116.60 (106.9–126.9)	0.46 (0.30–0.68)
	RTG-2	13.09 (11.88–14.37)	1.12 (0.78–1.61)

* The values in parentheses are 95% confidence intervals.

**Table 3:** IC_50_ values of finasteride and dutasteride in each cell line.

		IC_50_ Value	
		Finasteride (nM; 95% CI *)	Dutasteride (nM; 95% CI)
Human cell lines	LNCaP	241.0 (185.8–303.9)	1.26 (1.02–1.57)
	DU-145	308.5 (217.0–415.5)	3.83 (3.10–4.78)
	SW-13	213.5 (180.2–250.7)	4.75 (4.26–5.32)
Overexpression lines	SRD5A1	332.8 (260.9–424.8)	1.27 (0.76–2.06)
	SRD5A2	69.83 (33.65–133.3)	1.19 (0.96–1.47)
Fish cell lines	ZFL	142.4 (121.5–165.7)	7.33 (6.12–8.77)
	RTG-2	2667 (2394–2952)	13.19 (10.73–16.54)

* The values in parentheses are 95% confidence intervals.

**Table 4:** Percentage of amino acid identity of human, zebrafish, and rainbow trout 5ARs.

	**Zebrafish**		**Rainbow Trout**
	srd5a1		srd5a1
Human srd5a1	51.7		50.2
	**Zebrafish**		**Rainbow Trout**
	srd5a2a	srd5a2b	srd5a2a
Human srd5a2	52.3	42.4	50.2

Data were compared with human 5ARs amino acid sequence. The percentage of amino acid identity was compared using NCBI’s BLAST (http://blast.ncbi.nlm.nih.gov/Blast.cgi, accessed on 4 February 2021) and UniProt (http://www.uniprot.org, accessed on 4 February 2021). The sequences used for analysis are as follows (species, gene_GenBank GI ID): (Human, srd5a1_4507201, srd5a2_39812447); (Zebrafish, srd5a1_11549628, srd5a2a_62955375, srd5a2b_62202806); (Rainbow trout, srd5a1_1211289547, srd5a2_1211257249).

## Data Availability

The datasets generated during and/or analysed during the current study are available from the corresponding author on reasonable request.

## Sample Availability

Samples of the compounds are not available from the authors.
